# Fractal Characterization of the Mass Loss of Bronze by Erosion–Corrosion in Seawater

**DOI:** 10.3390/ma16103877

**Published:** 2023-05-22

**Authors:** Alina Bărbulescu

**Affiliations:** Department of Civil Engineering, Transilvania University of Brașov, 5, Turnului Street, 900152 Brașov, Romania; alina.barbulescu@unitbv.ro

**Keywords:** erosion–corrosion, cavitation, ultrasound, fractal dimension, multifractal

## Abstract

The fractal approach is one of the nondestructive techniques for analyzing corrosion’s effects on different materials. This article utilizes it to analyze the erosion–corrosion produced by cavitation on two types of bronze introduced into an ultrasonic cavitation field to investigate the differences between their behavior in saline water. The aim is to check the hypothesis that the fractal/multifractal measures significantly differ for the studied materials that belong to the same class (bronze) as a step in applying fractal techniques to distinguish between two materials. The study emphasizes the multifractal characteristics of both materials. While the fractal dimensions do not significantly differ, the highest multifractal dimensions correspond to the sample of bronze with Sn.

## 1. Introduction

Copper-based materials are frequently employed in many industries due to their behavior in different corrosive liquids [1,2,3,4,5]. The most common experimental procedures for the study of the behavior of such alloys are electrochemical methods [6,7,8] and synergy tests [3,9], noise recording [6], and electrochemical impedance spectroscopy [8,10]. The most used corrosive media are seawater [1], artificial NaCl solutions with different concentrations (3.5% in [2,10], other concentrations in [10]), NaCl, NaOH, and KOH [10]. Investigations of copper alloys have aimed at clarifying the unpassivation and corrosion mechanism of Ni-Al bronzes [1,4], explaining the corrosion–erosion in cavitation fields of Ni-Al bronze (and duplex stainless steel) utilized for propellers [3], describing crevice formation and propagation in Ni-Al samples kept for three years in natural seawater [5], and quantifying the variations in mechanical properties of samples of Cu alloys in various electrolytes [10]. It was also shown that the mechanical properties of Al-Fe bronzes increase when Sn is added.

After its creation [11] and development, the fractal theory became a valuable tool to characterize time series in natural sciences, engineering (geology, hydrology, electrical engineering, etc.) [12,13,14,15,16,17,18], for signal or image analysis, and for medicine [18,19,20,21,22,23,24,25,26]. Related to specific applications in mechanical engineering and material science, the fractal methodology aims at determining the characteristics of different materials during functioning cycles and the failure of elements built on them [27,28], characterizing the fractures that appeared in materials and their propagation [29,30,31,32,33,34], and analyzing the patterns of corrosion appearing after metal immersion in different media [35,36,37]. For example, in [30,38], fractal dimensions were employed to characterize the aspect of reinforced concrete samples after cracking and test the hypothesis that a correlation exists with the degree of damage suffered by the reinforcing bars. Spatial data were used in [39] to investigate the roughness of metal samples. Fractal and multifractal analysis were performed to investigate the results of corrosion or corrosion–erosion of steel and copper alloys [37,40,41,42,43,44].

Fractal analysis provides a measure of the complexity of an entire studied series or image. Multifractal analysis investigates the fractality at local zones, emphasizing the differences between the local dimensions in the global complexity of the study object.

Despite investigations of the mass loss of copper-based alloys having been performed [18,41,42,43] and the existence of multifractality applications in engineering, combined investigation (exploring the corroded surfaces of materials in an ultrasonic cavitation field) has been less reported [41]. Therefore, in this article, we continue the investigation from [8,41,42,43], aiming to determine the pattern of the mass loss of copper-based alloys from the viewpoint of fractal/multifractal theory. The goal is to check the hypothesis that the fractal/multifractal measures significantly differ for the two materials and compare the results with those obtained for a brass [41]. Accepting the above hypothesis will open up a new way to use this technique to distinguish between two classes of materials.

## 2. Materials and Methods

### 2.1. Experimental Setup and Materials

**Figure 1:**
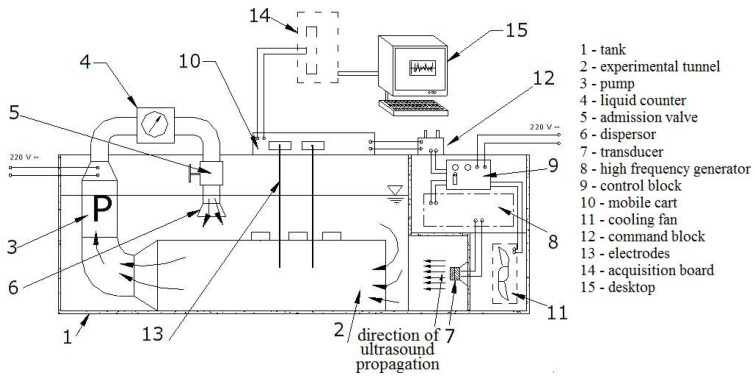
Setup for the cavitation study [8].

The experiments were performed using the setup from Figure 1. It consists of a tank for liquid (1) containing an experimental tube (2) that has an open end through which enters the fluid from the tank (1) and one end connected to the submersible pump (3). The cavitation is produced by a piezoceramic transducer (7) which enters into oscillation as a response to a high-frequency signal that it receives from the generator (8).

The control unit (9) controls the functioning of the generator (8)—at several levels of power—and of the cooler (11).

The mobile car (10) is powered and controlled by command block B (12). It is designed to move along the tank (1) and maintain the samples, in the case of a corrosion–erosion experiment (the actual case), or the measurement electrodes (13) that capture the electrical signal produced by cavitation (in the case of signals analysis). The electronic block which produces the high-frequency signals contains: a command block (9) with controls for access to the network and power adjustment, a high-frequency generator (8), a power transformer, radiators, and a cooler. The cavitation is produced by the oscillation of a ceramic transducer (7) that receives a signal from the high-frequency generator (8) that works at 18 kHz and different power levels (80, 120, or 180 W). The experiments presented here were performed without circulating the water, so the pump (3) was not switched on. The generator works at a voltage of 220 V and a frequency of 18 KHz. The electric powers developed at each level of the high-frequency generator (8) are 80, 120, and 180 W, which extends the range of parameters for conducting the experiments.

The materials used in the experime”t ha’ the following composition: Bronze 1 (Bz1): 4.05% Zn, 4.40% Pb, and 6.40% Sn in addition to Cu (83.09%), and Bronze 2 (Bz2): 4.40% Ni, 4.49% Fe and 9.85%, and Cu (80.54%).

The samples were introduced into the tank (1), which contained seawater kept at a constant temperature of 20 °C (utilizing the cooler (11)) for 1320 min. At each interval of 20 min, the samples were weighed after being cleaned. The values collected were used to build the time series of absolute mass variation per surface. It was achieved by computing the differences between the mass at a specific moment and the initial mass of the sample and then dividing the results by the sample’s surface area (measured before starting the experiment).

The seawater parameters were: 22.17 g/L NaCl, pH = 7, 0.31 g/L–${SO}_{4}^{2+}$, 0.051 mg/L Fe, 0.0033 mg/L Ni, and 6.27 meq/L total hardness. For details on the installation’s functioning, the reader may see [8].

### 2.2. Methodology

**Figure 2:**
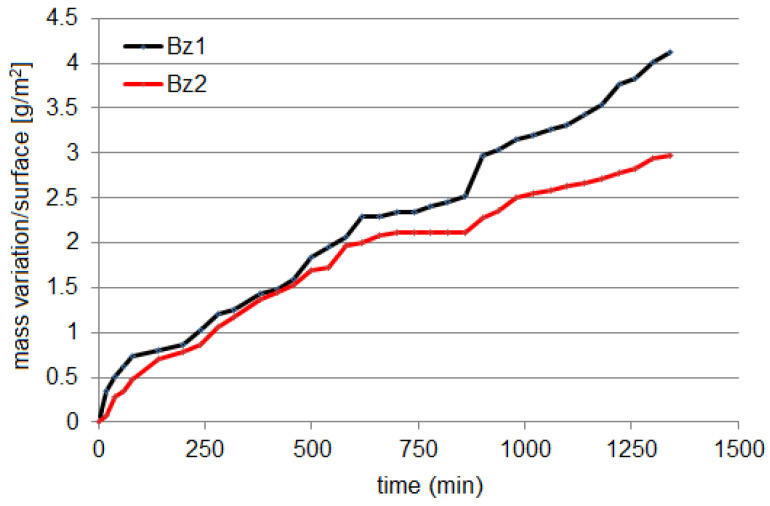
Time series of mass variation per surface.

The series we are working on are presented in Figure 2, and we shall refer to them as the Bz1 and Bz2 series.

The investigation was performed by fractal and multifractal analysis of the absolute mass loss curve. The background of the study is shortly presented in the following.

When introducing the fractal theory, Mandelbrot [11] found that the fractal dimension is a scale-invariant parameter of any geometrical object. A fractal object has the property that a part exhibits statistical similarity with other parts or the whole. Therefore, fractal objects obey scaling laws (power laws) at different scales. If a single-scale law can characterize an object, one refers to it as monofractal; otherwise, one calls it multifractal [11,44].

Fractal objects are generally characterized by various dimensions. Some of them, presented in the following, were used in this study.

The box-count dimension of a bi-dimensional object in a plane is calculated by [45]:

(1) $$D_{BC}=\underset{\varepsilon \to o}{\lim}N(\varepsilon )/log(1/\varepsilon )$$

where *N*(*ε*) (*ε* > 0) is the minimum number of squares having a side of length *ε* used for covering the object.

Practically, to estimate $D_{BC},$ the object is covered by boxes that are divided in each step into several smaller boxes, keeping only those containing at least a point. $D_{\mathrm{BC}}$ is computed as the slope of the line fitted (by the least squares method, on the log–log scale) to the number of boxes vs. the side dimension. In our experiments, the boxes were also rotated by angles of 12 degrees, and the resulting dimensions in all the experiments were averaged. The dimensions are reported together with the standard deviations, their minimum and maximum values, and the dimension with the highest R^2^ square in a specific set of experiments.

The Hall–Wood [46] estimator of the fractal dimension, $D_{HW},$ takes into account at a certain scale the sum of areas, $A\left(\varepsilon \right),$ of all boxes intersecting the least squares line fitted when computing (1). Therefore,

(2) $$D_{HW}=2-\underset{\varepsilon \to o}{\lim}A(\varepsilon )/log(\varepsilon )$$

The *p-order variogram estimator*, proposed in [39] and improved by Genton [47], is based on the computation of the moment estimators of the structure function of a stochastic process, at different lags *d*, $\hat{V_{d}}$. Therefore, the variogram estimator is the slope of the least squares regression line of log ($\hat{V_{d}}$) vs. log(*d*) [47,48]. Particular cases are the madogram (*p* = 1) and variogram (*p* = 2).

Other dimensions used in fractal analysis, derived from the box-count dimension, are the *correlation dimension* ($d_{corr}$) and *capacity dimension* ($d_{cap}$) [49]. The first one is obtained when the figure is covered by circles or squares, and the mean number of points inside each circle or square is considered as *N*(ε) in (1). The second one is computed by:

(3) $$d_{cap}=-\underset{\begin{array}{c} \varepsilon \to 0\\ \varepsilon >0 \end{array}}{\lim}\frac{ln(N(\varepsilon ))}{ln(\varepsilon )},$$

Utilizing the values of the normalized probability that the *i*th cell of the cover is not void ($P_{i}\left(\varepsilon \right))$, the *information dimension* ($d_{inf}$) is given by [49]:

(4) $$d_{inf}=\underset{\begin{array}{c} \varepsilon \to 0\\ \varepsilon >0 \end{array}}{\lim}\frac{\sum_{i=1}^{N_{\varepsilon }}P_{i}\left(\varepsilon \right)ln(P_{i}\left(\varepsilon \right))}{ln(\varepsilon )}.$$

Multifractal detrended fluctuation analysis (MFDFA), introduced by Kantelhardt et al. [50], can demonstrate a time series’ multifractal characteristics. It is used in this article to analyze the series from Figure 2. The method is shortly described in the following [50,51,52,53].

Consider a time series ${\left(y_{t}\right)}_{t=1,n}$.

1. Compute the centered series by subtracting the mean,$\bar{y}$, from each $y_{t}$, t=1,n:

(5) $$z_{i}=\sum_{t=1}^{\tau }(y_{t}-\bar{y}),\tau =\bar{1,n}$$

2. Divide $z_{i}$ into $n_{s}$ segments with the same length (s), nonoverlapping, and such that every two consecutive segments have only a common point—the end of one segment and the beginning of the next. Because generally n is not divisible by s—the segmentation length—and some elements at the end of the series (5) are not used, the series partition is also performed starting from $z_{n}$ to $z_{1}.$ Therefore, $2n_{s}$ segments are obtained.

3. Compute the least squares polynomial trend (of the first, second, or third degree) for all the subseries built in the previous step and the corresponding detrended sub-series, $x_{t}\left(i\right)(1<i<s)$.

4. Compute the variances:

(6) $$F^{2}(s,t)=\frac{1}{s}\sum_{i=1}^{s}x_{t}^{2}(i),t=\bar{1,2n_{s}}$$

5. Calculate the *q*-order fluctuation function as follows:

(7) $$F_{q}\left(s\right)=\left\{\begin{array}{c} {\left[\frac{1}{2n_{s}}\sum_{t=1}^{2n_{s}}{\left(F^{2}(s,t)\right)}^{q/2}\right]}^{1/q},if\;q\neq 0\\ exp\left[\frac{1}{4n_{s}}\sum_{t=1}^{2n_{s}}\ln\left(F^{2}(s,t)\right)\right],if\;q=0 \end{array}\right.$$

When *q* = 2, the detrended fluctuation analysis (DFA) [54] is retrieved. To determine the variation of $F_{q}\left(s\right)$ on s, stages 2–4 must be repeated for different values of *s*.

6. Analyze the log($F_{q}\left(s\right)$) vs. log(*s*) chart for each *q* to determine a power law dependence:

(8) $$F_{q}\left(s\right)∼s^{h_{q}}$$

where $h_{q}$ is the generalized Hurst exponent that must be found.

For multifractal series, $h_{q}$ depends on *q*: when *q* increases, $h_{q}$ monotonically decreases. For the monofractal series, there is no dependence of $h_{q}$ on *q*.

In the case of the stationary series, there is a direct relationship between $h_{q}$ and the scaling exponents $\tau_{q}$ defined in standard fluctuation analysis [55] and linked to the partition function, $Z_{q}$, by the equation:

(9) $$Z_{q}\left(s\right)=\sum_{k=1}^{n/s}{\left|p_{s}(k)\right|}^{q}\sim s^{\tau_{q}},$$

where $p_{s}(k)$ is the *k*th segment box probability [55,56,57].

Therefore, the following equation linking $\tau_{q}$ and $h_{q}$ can be derived [58]:

(10) $$\tau_{q}=qh_{q}-1$$

In the case of multifractality, there is a nonlinear dependence between $\tau_{q}$ and *q* (more accentuated when the nonlinearity is higher), whereas in the monofractality case, the dependence is linear [59].

The series multifractality can be also analyzed utilizing the singularity spectrum *f*(α), obtained after a Legendre transformation of $\tau_{q}$, such as in [57]:

(11) $${\alpha =d\tau }_{q}/dq\;and\;f(\alpha )=q\alpha -\tau_{q}$$

α is named the Hölder exponent.

In the monofractality situation, a unique α characterizes the entire series, so $f\left(\alpha \right)$ is formed by a single point. In a multifractal situation, many α-values characterize the series (each for a sub-series), giving birth to the $f\left(\alpha \right)$—spectrum.

The spectrum width is computed by:

(12) $${∆\alpha =\alpha }_{max}-\alpha_{min}$$

The higher the value of ∆α, the more accentuated the multifractal character is.

Another measure of multifractality is the *generalized multifractal dimension* (*Renyi’s dimension*) [58,59], $D_{q},$ whose dependence on $\tau_{q}$ is given by:

(13) $$\tau_{q}=\left(q-1\right)D_{q}\Leftrightarrow D_{q}=\frac{1}{q-1}\tau_{q}.$$

The monofractality is characterized by a linear (almost horizontal) shape of the plot of $D_{q}$ as a function of *q*. In the multifractal case, the plot’s shape is inverse sigmoid.

The analysis was performed using the R 4.2.3 (https://www.r-project.org/) and Fractalyse (https://sourcesup.renater.fr/www/fractalyse/, accessed on 26 April 2023) software. First, the mass loss series was introduced into the first two columns of a .csv file. Then, they were read by R (as data frames). The following libraries were uploaded: “tseries,” “fractaldim,” and “MFDFA”. The first one permits the transformation of each series into a time series using the “as.ts()”command. The second one was employed to determine the box-count and Hall–Wood estimators, variogram, and madogram. The third one permits multifractal analysis. The information, capacity, and correlation dimensions were computed using Fractalyse.

## 3. Results and Discussion 

### 3.1. Models of the Data Series

**Figure 3:**
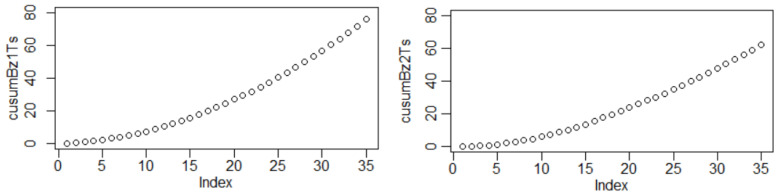
CUSUM diagrams for the samples of (**left**) Bz1 and (**right**) Bz2.

According to Figure 1, the mass loss variation per surface was the highest for Bz2 at all experimental stages. The same observation can be extracted from the cumulated sum (CUSUM) charts (Figure 3). The mass loss ($∆m_{t}$) per surface (*S*) in time (*t*) can be described by Equation (10) for Bz1 and Equation (11) for Bz2:

(14) $$∆m_{t}/S=0.1444+0.1126t\;(R^{2}=0.9957),$$

(15) $$∆m_{t}/S=-0.3068+0.1968t-0.005t^{2}-0.00006t^{3}(R^{2}=0.9936).$$

A linear model with R^2^ above 0.99 can also be fitted to the second series without a significant accuracy loss.

### 3.2. Fractal Analysis of the Sample Surface after Corrosion

**Figure 4:**
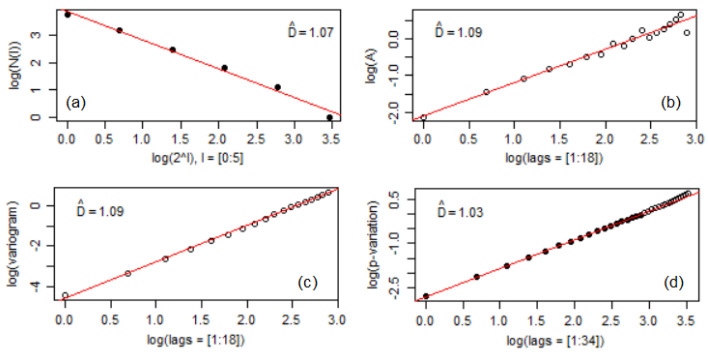
Fractal dimensions for Bz1 series: (**a**) $D_{BC}$, (**b**)$D_{HW}$, (**c**) variogram, (**d**) madogram.

**Figure 5:**
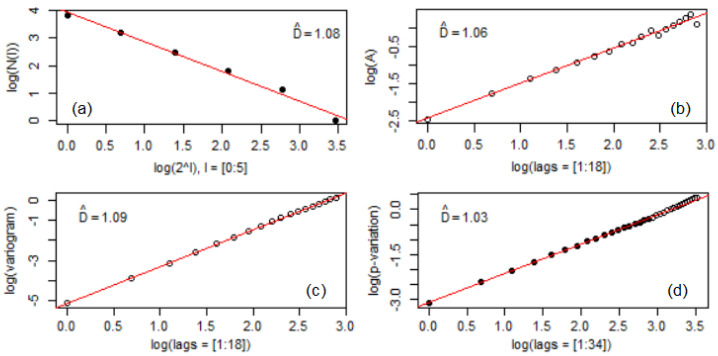
Fractal dimensions for Bz2 series: (**a**) $D_{BC}$, (**b**)$D_{HW}$, (**c**) variogram, (**d**) madogram.

The values of the fractal dimensions computed by the box-count estimator, Hall–Wood estimator, variogram, and madogram methods are presented in Figure 4 and Figure 5 (obtained by using the R software). No significant differences between these dimensions are noticed. The highest difference is between the variograms’ values (Figure 4b and Figure 5b). All the values are between 1.03 and 1.09. At this stage, no significant difference can be found regarding the fractal properties of the two series.

### 3.3. Multifractal Analysis of the Sample Surface after Corrosion

**Figure 6:**
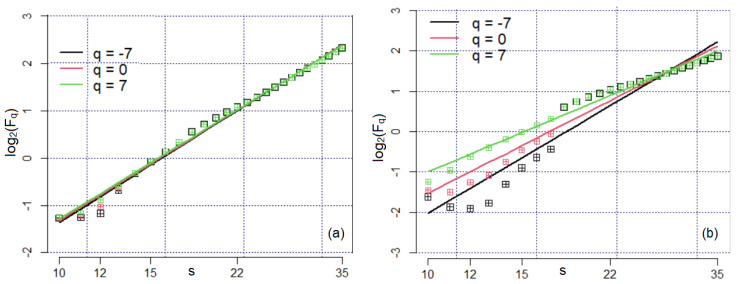
Charts of $F_{q}$ vs. *q* in ${log}_{2}-{log}_{2}$ scale for (**a**) Bz1 and (**b**) Bz2 series.

According to the theory from the previous section, to determine the exponents $h_{q}$ from (8), one must study the charts of ${log(F}_{q}(s))$ vs. log(s) for different values of *q.* Such charts are shown in Figure 6 for three values of *q.* In Figure 6a, one may notice one obvious direction, with no significant changes in the slope in all cases. Only a few points have a slight deviation from the trend lines. So, a very low multifractal character is noticed. In the plots from Figure 6b, there are some deviations of the computed values—squares—from the linear trend lines, which are more accentuated for *q* = 0 and *q =* 7. For example, for *q =* −7, a decreasing trend is noticed for *s* from 10 to 12, followed by an increasing trend from 12 to 18 and another slope change from 18 to 35. These breakpoints indicate a multifractal character of the Bz2 series, showing the scaling presence at any *q*. The linear trend fitted for $F_{q}(s)$ has a corresponding R^2^ above 0.98.

**Figure 7:**
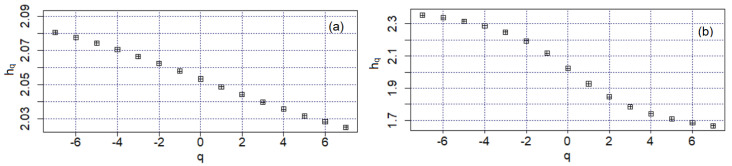
Hurst exponents in MFDFA for (**a**) Bz1 and (**b**) Bz2.

Figure 7 presents the Hurst exponents in MFDFA. Both $h_{q}$ series monotonically decrease when *q* increases, indicating multifractal behavior for both series. Still, one should consider all of the information extracted from the analysis before deciding on this character.

**Figure 8:**
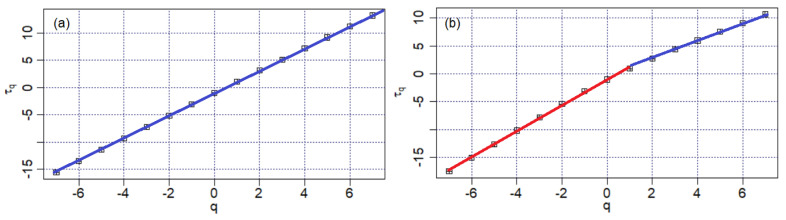
The charts of the scaling exponent, $\tau_{q},$ for (**a**) Bz1 and (**b**) Bz2 series.

Figure 8 contains the graphical representation of $\tau_{q}$ with respect to *q* for *q* $\in \left[-7,7\right]$.

The first chart (Figure 8a) has a linear shape. The second one (Figure 8b) presents a slight slope change. The two segments with different slopes are represented with different colors. Therefore, the first chart indicates a possible monofractal character of the Bz1 series, whereas the second one indicates the multifractality of the Bz2 series. To sustain or reject these assertions, the *f*(α)—spectrum was computed in each case.

**Figure 9:**
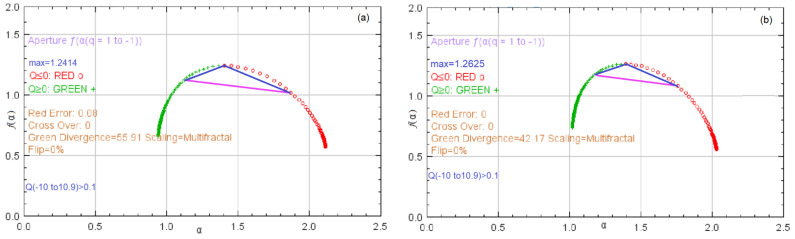
The $f\left(\alpha \right)$—spectrum for (**a**) Bz1, and (**b**) Bz2 series.

Figure 9a shows the *f*(α)—spectra for the two series, calculated by averaging the values from all experiments. In the first case, the max *f*(α) = 1.2412, while in the second one, it is 1.2625. Comparisons between the aperture lengths are presented in Table 1. Smaller aperture lengths are determined for the second series. In both cases, the suggested scaling is multifractal.

The multifractal dimension is 1.1737, with the standard deviation SD = 0.0856. The minimum multifractal dimension in all experiments is 1.3148, and that with the highest R^2^ = 0.9776 is 1.0958; the corresponding SD = 0.1727. The computation of the multifractal dimension using the *f*(α)-spectrum results in 1.1558, with R^2^ = 0.9928 and SE = 0.1027, for Bz2. The correlation dimension, capacity dimension, and information dimension were, respectively:

${D_{0}=d}_{cap}=1.2414$ for Bz1 (1.2625 for Bz2),

${D_{1}=d}_{inf}=1.1244$ for Bz1 (1.1709 for Bz2),

${D_{2}=d}_{cor}=1.0664$ for Bz1 (1.1262 for Bz2).

The highest dimensions correspond to the second sample.

Comparisons of the previous results with those drawn from the analysis of a brass sample [41] subjected to the same conditions indicate that:

- The fractal dimensions of all series do not significantly differ.
- The lowest capacity dimension corresponds to the brass series −$D_{0}$ = 1.22.
- The Bz1 and Bz2 series have a multifractal character that is not evident for the brass series, for which the f-alpha shape indicates multifractality, whereas that of *D_q_* indicates a monofractal character of this series.

In this study, the fractal analysis results in a measure of the whole mass loss process, quantifying its global complexity. The multifractal study investigates the fractal properties of different subseries of the series. It indicates that the data series has various dimensions at a local scale, which differs from the global one, so the analyzed process has different behaviors at local and global scales. The fractality of some copper alloys’ behavior has been investigated in studies related to electrochemical corrosion [1,2,3,4,5,6,7] and signal analysis [18]. Also, multifractal analysis of corroded surface images has been performed for different materials [38,60,61,62]. Still, these studies have been performed using a smaller number of tools, among which are R/S rescaled analysis and MFDFA [50]. Extended analyses on the mass loss of copper alloys have not been performed using both fractal and multifractal methods, even if the mass loss in time of copper alloys has not been studied in terms of its relationship with image analysis [41]. The current study and [41] represent a departure point for the fractal analysis of these materials’ behavior in a cavitation field in seawater by fractal and multifractal approaches. Whereas in [38], the multifractality index was utilized to distinguish the corrosion type, the novelty of the present study is that fractal dimensions were employed to distinguish the mass loss of different copper alloys.

## 4. Conclusions

In this paper, we investigated the fractal characteristics of two series of mass loss of two samples of bronzes subjected to ultrasound cavitation. It was shown that the fractal dimensions vary between 1.03 and 1.09, with no significant difference between the series. The values of the multifractal dimensions indicate a multifractal character of both series, which is more accentuated for the second one. Comparison with the brass sample behavior shows that the applied technique can be used to characterize the behavior of different materials in a cavitation field.

This analysis has the advantage of no restrictions on the liquid or material used in the experiment. Other aspects should be studied, like the dependence between the fractal variation and different experimental stages or building a multivariate model to reflect the dependence of the fractal characteristics on the material’s composition and structure.

The following summarizes the research’s importance:

- Experiments have been conducted using an installation designed by us for the study of ultrasound influence on materials in different liquids.
- The bronzes used in the study were analyzed only in some of our research from the viewpoint of their behavior in the cavitation field, but no study has been carried out to describe the mass loss using fractal techniques.
- The models of mass loss of some materials in general, and in a cavitation field especially, together with fractal dimensions can distinguish between different materials’ behavior.
- Investigation of the materials’ mass loss using the multifractal technique leads to determining the pattern process and the changes that appear when the process advances.
- It was proved that the multifractal character of the mass loss of the brass sample cannot be sustained, whereas the bronzes’ series have no such issue.

The study will be developed with the analysis of some composites (copper materials) to find a classifier of copper based-alloys using fractal dimensions.

## Figures and Tables

**Table 1:** Apertures in the MFDA of Bz1 and Bz2 series.

	Aperture Length Q (1 to 0)	Aperture Slope Q(1 to 0)	Aperture Length Q (0 to −1)	Aperture Slope Q(0 to −1)	Aperture Length Q(1 to −1)	Aperture Slope Q(1 to −1)
Bz1	0.3061	0.4135	0.5135	−0.4764	0.7536	−0.1392
Bz2	0.2400	0.4134	0.4065	−0.4870	0.5936	−0.1469

## Data Availability

Not applicable.
